# Natural phenolics greatly increase flax (*Linum usitatissimum*) oil stability

**DOI:** 10.1186/s12896-015-0178-0

**Published:** 2015-06-30

**Authors:** Karolina Hasiewicz-Derkacz, Anna Kulma, Tadeusz Czuj, Anna Prescha, Magdalena Żuk, Magdalena Grajzer, Marcin Łukaszewicz, Jan Szopa

**Affiliations:** Faculty of Biotechnology, University of Wroclaw, Przybyszewskiego 63/77, 51-148 Wroclaw, Poland; Linum Foundation, Plac Grunwaldzki 24a, 50-363 Wroclaw, Poland; Department of Food Science and Dietetics, Wroclaw Medical University, Borowska 211, 50-556 Wroclaw, Poland; Department of Genetics, Plant Breeding and Seed Production, The Faculty of Life Sciences and Technology, Wroclaw University of Environmental and Plant Sciences, Pl. Grunwaldzki 24A, 53-363 Wrocław, Poland; Faculty of Biotechnology, University of Wroclaw, Fryderyka Joliot-Curie 14a, 50-383 Wrocław, Poland

**Keywords:** Flaxseed oil, Oil stability, Antioxidant activity, Phenolic compounds, Radical scavenging activity

## Abstract

**Background:**

Flaxseed oil is characterized by high content of essential polyunsaturated fatty acids (PUFA) promoted as a human dietary supplement protecting against atherosclerosis. The disadvantage of the high PUFA content in flax oil is high susceptibility to oxidation, which can result in carcinogenic compound formation. Linola flax cultivar is characterized by high linoleic acid content in comparison to traditional flax cultivars rich in linolenic acid. The changes in fatty acid proportions increase oxidative stability of Linola oil and broaden its use as an edible oil for cooking. However one of investigated transgenic lines has high ALA content making it suitable as omega-3 source. Protection of PUFA oxidation is a critical factor in oil quality. The aim of this study was to investigate the impact of phenylpropanoid contents on the oil properties important during the whole technological process from seed storage to grinding and oil pressing, which may influence health benefits as well as shelf-life, and to establish guidelines for the selection of new cultivars.

**Methods:**

The composition of oils was determined by chromatographic (GS-FID and LC-PDA-MS) methods. Antioxidant properties of secondary metabolites were analyzed by DPPH method. The stability of oils was investigated: a) during regular storage by measuring acid value peroxide value p-anisidine value malondialdehyde, conjugated dienes and trienes; b) by using accelerated rancidity tests by TBARS reaction; c) by thermoanalytical - differential scanning calorimetry (DSC).

**Results:**

In one approach, in order to increase oil stability, exogenous substances added are mainly lipid soluble antioxidants from the isoprenoid pathway, such as tocopherol and carotene. The other approach is based on transgenic plant generation that accumulates water soluble compounds. Increased accumulation of phenolic compounds in flax seeds was achieved by three different strategies that modify genes coding for enzymes from the phenylpropanoid pathway. The three types of transgenic flax had different phenylpropanoid profiles detected in oil, highly increasing its stability.

**Conclusions:**

We found that hydrophilic phenylpropanoids more than lipophilic isoprenoid compounds determine oil stability however they can work synergistically. Among phenolics the caffeic acid was most effective in increasing oil stability.

## Background

Flax (*Linum usitatissimum* L) is an annual plant that is widely distributed in the temperate climate zone. Its three main products are seeds, oil and fibers. For years, flax seeds have been recommended for the human diet, because of their high content of components that are beneficial for human health. They contain a relatively high quantity of polyunsaturated fatty acids (PUFA), secoisolariciresinol diglucoside [1], phenolic acids and flavonoids. Flax oil is valuable due to its high quantity of essential PUFA: α-linolenic acid (ALA) and linoleic acid (LA). Today flax oil is valuable due to its high quantity of essential PUFA: α-linolenic acid (ALA) while traditionally flax oil was valued for its high quantity o of essential PUFA. Linola flax oil with reduced ALA content may serve as valuable replacement of sunflower and corn oil for culinary purposes. The climate in Poland and northern Europe is more suitable for Linola than for sunflower and maize cultivation [2]. However, in one of the transgenic lines used in this study, the content of ALA increased significantly due to genetic modification making it suitable for use as ω-3 diet supplement [3]. It has been widely proven that a high level of ALA in the diet can reduce the risk of cancer [4] and cardiovascular diseases [5] and decrease the production of arachidonic acids and other pro-inflammatory eicosanoids [6]. The simultaneous consumption of ω-3 and ω-6 acids in a proper ratio (1/1 to 4/1) is essential in cancer prevention and inflammation reduction. The current western diet typically contains an excess of ω-6 acids (around 15/1 in most areas), but consumption of oils with a high level of ω-3 fatty acids such as flax oil could be a good solution [7].

Protection from lipid oxidation is critical factor in oil quality for consumption as peroxidation of PUFA may lower cell viability [8]. Degradation of fatty acids starts during seed storage, despite containing antioxidants such as tocochromanols (tocopherols, tocotrienols, plastochromanol-8), lutein [9–12] and phenolic compounds. The results of in vitro experiments showed that hydrophilic phenolic compounds were more effective than hydrophobic tocopherol in preventing fatty acid oxidation in the oil [13] and this phenomenon is known as a polar paradox [14]. The better efficacy of phenolics then lipophilic antioxidants in oils has been explained by the location of polar compounds at the interface of different association colloids, formed from e.g. hydroperoxides and aldehydes.

However, phenolic compound contents are minor and thus far have been only poorly identified in flax oil. Plant phenolics are a diverse group of biochemical compounds, the majority of which originate from the phenylpropanoid pathway. Many possible modifications catalyzed by specific enzymes give a high diversity of phenylpropanoid compounds which may be in the last step glycosylatated, giving them greater stability and higher water solubility. In flax, lignans are common in the seeds, with the most abundant being secoisolariciresinol diglucoside. There might also be found cinnamic acid derivatives, such as p-coumaric acid, caffeic acid, ferulic acid and their glycosylated or esterified forms, and the flavonoids pelargonidin, cyanidin, delphinidin, herbacetin, kaempferol, luteolin and apigenin, and their glucosides, such as orientin, isoorientin, vitexin, isovitexin, vicenin and lucenin [15].

Three types of transgenic flax plants (based on Linola variety) were previously generated with increased phenylpropanoid contents, protecting plants from biotic and abiotic stresses [16]. All three flax types (W92, W86, GT) accumulated a higher level of phenylpropanoid components, resulting in a higher antioxidant capacity, and thus higher resistance against pathogenic *Fusarium* infection and higher plant productivity [3, 17–19]. The three types of transgenic flax differ in their accumulation of particular components. In the first type (W92), the simultaneous expression of chalcone synthase (CHS), chalcone isomerase (CHI) and dihydroflavonol-4-reductase (DFR) cDNAs resulted in a significant increase in the content of phenylpropanoid compounds (lignans, flavonoids and phenolic acids) in the plant products, including the seeds [17]. In the second type (W86), down-regulation of endogenous CHS again resulted in accumulation of phenylpropanoid compounds, but it was mainly hydrolysable tannins in this case [3]. A similar effect was obtained in the case of the third type (GT), which overproduces glycosyltransferase, resulting in higher accumulation of mainly glucoside derivatives of phenolic acids in the seeds [18, 20].

The aim of this study was to investigate the impact of phenylpropanoid contents on the oil properties important throughout the technological process – seed storage, grinding, and oil pressing – which may influence probiotic properties and shelf-life, and to establish guidelines for the selection of new cultivars. We identified and measured the phenylpropanoid levels in the oil and correlated their quantity with their antioxidant capacity and stability. An increased quantity of phenolics was found in the oil from the transgenic plants, and there was a detectable increase in the antioxidant capacity and oil stability. To the best of our knowledge, this is the first complete report with detailed identification and quantification of water-soluble phenolics in flax oil and demonstrating a correlation between oil stability and the level of secondary metabolites of both hydrophilic and hydrophobic nature.

## Results

### Oil parameters

The average yield of flax oil pressed from the control and transgenic seeds was 29 % of the seed weight. There was no difference in the yield of oil from the control and transgenic seeds. Only a slight difference (determined visually) in the color of the oil from the control and transgenic plant seeds was detected. W86 oil was slightly darker and GT oil was slightly lighter than the control (Linola) and W92 oil. The fatty acid profiles of the GT, W92 and W86 oil were compared with oil from non-transgenic control flax of Linola variety (Table 1). Modifications of the phenylpropanoid pathway resulted in variations of FA profiles. W86 oil showed almost a 10-fold increase in the ω-3 level. In contrast, an over 7 % increase in the level of ω-6 was detected in W92. The observed variations of PUFA content will affect oil stability.

**Table 1:** GC-FID analysis of fatty acid contents in flax oil from control (Linola) and three transgenic lines (GT, W92, W86)

Flax oil	Fatty acids content (g/100 g oil)					
	Palmitic acid (C16:0)	Stearic acid (C18:0)	Oleic acid (C18:1)	Linoleic acid (C18:2)	α-Linolenic acid (C18:3)	Σ unsaturated acids
Control	5.42 ± 0.53	3.95 ± 0.12	16.70 ± 0.25	74.09 ± 1.84	2.68 ± 0.08	93.48
GT	5.64 ± 0.13	2.98 ± 0.06*	13.94 ± 0.27*	76.24 ± 1.85	2.03 ± 0.07*	92.22
W92	6.67 ± 0.03	3.30 ± 0.01*	14.56 ± 0.25*	81.72 ± 1.82*	2.53 ± 0.08	98.81
W86	5.21 ± 0.24	3.86 ± 0.21	16.29 ± 0.72	49.57 ± 1.85*	20.54 ± 0.96*	86.41

The results are the average of 5 repetitions ± SD; ^*^statistically significant data (*p* < 0.05)

### Shelf life stability of oils

**Fig. 1:**
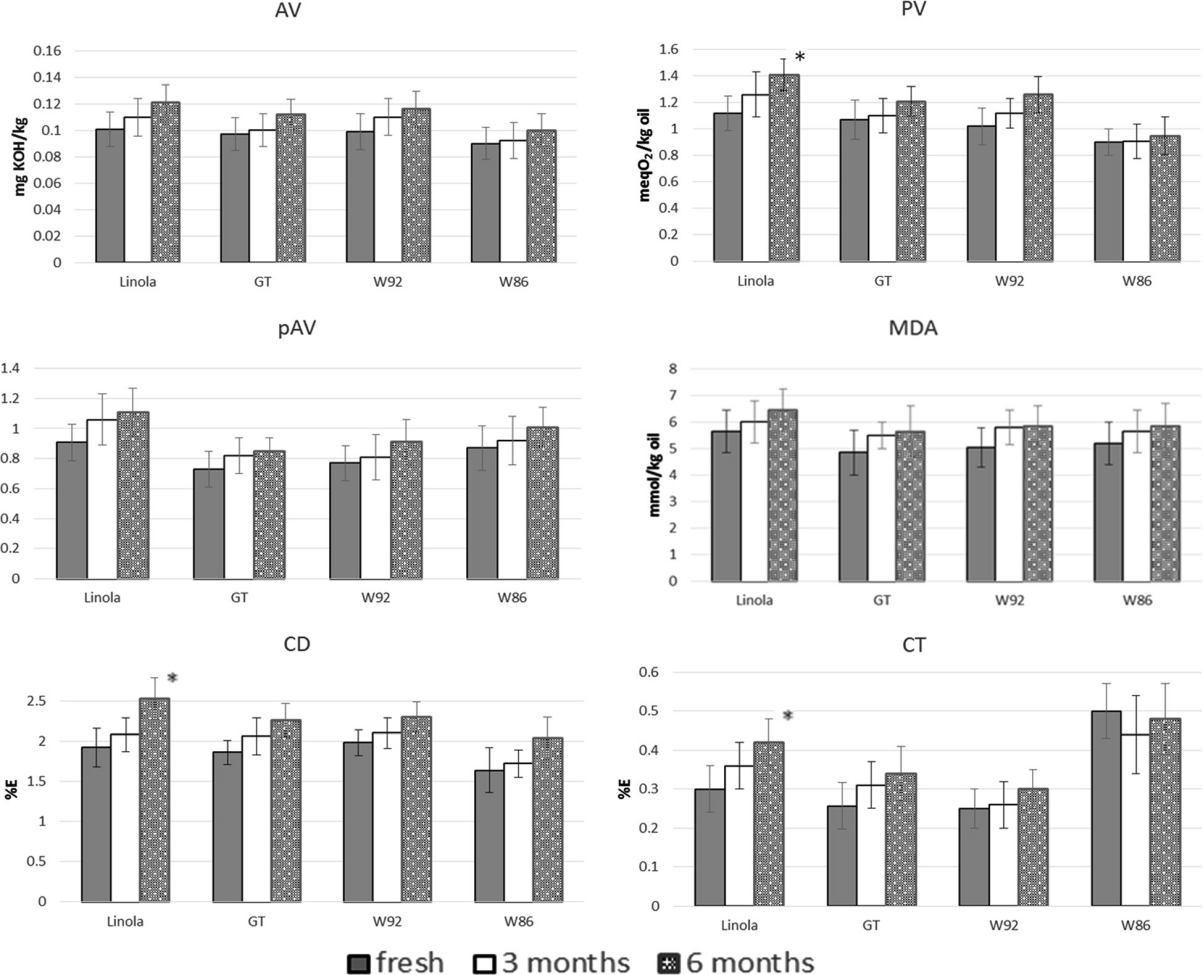
Oxidation parameters measured in fresh oils and after 3 and 6 months of storage at room temperature (20 ± 10C) AV-acid value, PV-peroxide value, pAV-p-anisidine value , MDA- malondialdehyde content, CD- conjugated dienes ,CT- conjugated trienes

Oxidative stability of cold-pressed flax oils throughout their shelf life (at room temperature) was determined by the acid value (AV), peroxide value (PV), *p*-anisidine value (pAV) as well as the contents of malondialdehyde (MDA), conjugated diene (CD) and triene (CT) measured in the fresh oils, and after 3 and 6 months of storage (Fig. 1).

AV measures the content of free fatty acids formed upon the hydrolytic degradation of lipid molecules, thus contributing to the reduction of the shelf life of the oil. The acid value of each of oils in each of the indicated periods of storage was within the limit of up to 4 mg KOH/g of oil, according to the Codex Alimentarius Commission standard for cold-pressed and virgin oils [21]. A statistically insignificant trend toward a higher free fatty acid contents in all studied oils during shelf life were observed and the obtained values did not exceed 0.121 mg KOH/kg oil in Linola.

PV defines the content of lipid peroxides and hydroperoxides in oils formed in the initial stages of auto- and photo-oxidation. In the tested oils, slightly increased PV was observed after 3 and 6 months, and a statistically significant difference was reported only in Linola (up to 25.8 %) at the end of its shelf life. Generally PV were lower than 1.5 meq O2/kg during whole shelf life period in all studied oils and none of them exceeded the recommended limit for cold-pressed oils of 15 meq O2/kg.

Hydroperoxides, the primary oxidation products of PUFA, react with oxygen to form MDA which may contribute to off-flavor of oxidized oils. The contents of MDA and other aldehyde which form adducts with TBA were evaluated in oils using TBARS method. MDA equivalents were not formed at a significantly higher levels after 3 and 6 months of storage of Linola and transgenic oils.

The levels of aldehydes in oils were also determined using the *p*-anisidine test. *p*AV enables us to measure the level of non-volatile aldehydes, especially 2,4-dienals and 2-alkenals in stored oils [22]. As in case of MDA equivalent measurements, the lowest contents of aldehydes expressed as *p*AV were shown in fresh and stored GT oil and the highest in Linola, with an average 22.4 % increase in the sixth month of shelf life. Nevertheless, this increment was not statistically significant.

The formation of hydroperoxides from PUFA may result in double bond isomerisation. The determination of conjugated dienoic and trienoic fatty acid derivatives (CD and CT) enable definition of the oxidation state of an oil, in addition to PV. Analogously to changes of PV in oils during storage the highest and statistically significant increase of CD (31.8 %) and CT (40.0 %) contents were found in Linola stored 6 months. The relatively high initial level of CT in W86 resulted from the increased ALA percentage in this oil, while the formation of trienoic fatty acid derivatives during storage was not observed, and was even slightly decreased.

The oil stability parameters (especially AV, PV, *p*AV) are commonly used in shelf life assessment and are included in quality definition of oil as a food product. However these parameters do not supply complex information about oxidation product occurrence at different extents of oil deterioration and give limited data on the chemical kinetics of oxidation. The thermoanalytical methods e.g. differential scanning calorimetry (DSC) are nowadays used for assessing the autooxidation of oils. DSC analyses enable continuous monitoring of the total thermal effect of lipid accelerated oxidation [23] and moreover the prediction of expected reaction rates at low temperatures. The results obtained during DSC (Table 2) analyses allowed us to rank oils in terms of their oxidative stability and report the oxidation induction time at 20 °C-imitating the start of lipid oxidation during the shelf life [24]. The highest oxidation onset time and the longest propagation period in all studied isothermal runs was shown for W92 oil. Oxidation onset time indicates the set point of the primary products formation from PUFA oxidation where propagation time can be consider as a resistance to auto-oxidation. However W86 was found to have the longest induction time reported as a shelf life at 20 °C among studied oils. Induction time is a period of time that no change in the heat flow occurs and is often considered as a measurement of oil stability [25].

**Table 2:** Kinetic parameters of DSC study and calculated oxidation induction time for shelf life

Oil	Isothermal T (°C)	t_0_ (min)	t_max_(min)	Regression equation_a_	R^2^_b_	Reported t_0_ or shelf life at 20 °C (d)
Linola						
	100	170.7	287.1	Log (k) = 0.0211 T-4.3479	0.9987	5.9
	110	108.3	183.7			
	120	64.6	114.5			
GT						
	100	162.5	229.9	Log (k) = 0.0237 T – 4.5500	0.9633	8.3
	110	78.4	128.4			
	120	54.6	88.4			
W 92						
	100	200.1	424.3	Log (k) = 0.0240 T – 4.7354	0.9574	12.5
	110	140.8	297.9			
	120	66.1	210.5			
W 86						
	100	118.5	270.8	Log (k) = 0.0277 T – 4.8907	0.9346	15.1
	110	83.9	186.7			
	120	33.0	120.5			

^a^based on (Tan et al. [24])

^b^Correlation determination

*k* kinetic rate constant

### Accelerated rancidity tests of oils – TBARS reaction

TBARS method is very useful and simple method allowing the assessment of susceptibility to oxidation (T1) of native and antioxidant-spiked oil via measurements of the MDA level – a stable secondary product of PUFA peroxide decomposition. T1 of oils from control and transgenic plant seeds was evaluated via the TBARS (thiobarbituric acid reactive substances) method. TBARS assay results are reported as the equivalent of malondialdehyde (MDA), a compound that results from decomposition of polyunsaturated fatty acid lipid peroxides.

**Fig. 2:**
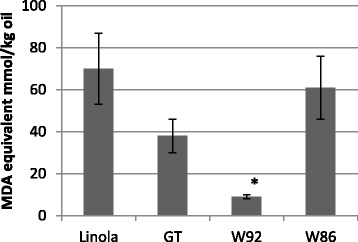
Content of fatty acid oxidation product in oils from control (Linola) and transgenic plants (GT, W92, W86) heated for 30 min at 140 °C (T1). Oxidation was measured using the TBARS method and data were expressed as MDA equivalent (g/100 g oil). The results are the average of 5 repetitions ± SD; ^*^statistically significant data (*p* < 0.05)

**Fig. 3:**
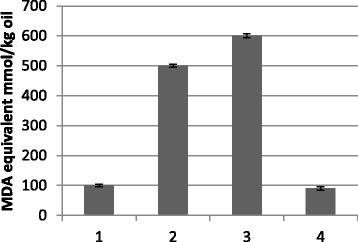
Content of fatty acid oxidation product in oil from control (Linola) flax plants (1) supplemented (2–4) with α-linolenic acid (18:3) standard heated for 30 min at 140 °C (T1), measured using the TBARS method, with data expressed as MDA equivalent (g/100 g oil). 1, no added α-linolenic acid; 2, supplemented with half of the α-linolenic acid (11.29 g/100 g oil) quantity found in W86 oil; 3, supplemented with α-linolenic acid in the amount found in W86 oil (20.54 g/100 g oil); 4, W86 oil. The results are the average of 5 repetitions ± SD, ^*^statistically significant data (*p* < 0.05)

The susceptibility to oxidation (T1) was measured by heating oil at 140 °C for 30 min in air atmosphere. The experimental conditions were chosen to obtain sufficiently high level of TBARS in oils, which allowed us to precisely show the influence of even slight differences in concentrations of antioxidants on the formation of these compounds. These conditions of TBARS test were also used by Lukaszewicz et al. to study the susceptibility of different flax cultivars to oxidation (Lukaszewicz 2004). The TBARS measurements showed a decrease in the level of oxidation products by 10.21 %, 50.93 % and 86.53 % for W86, GT and W92 oil, respectively, compared to the control (Fig. 2). Of note, in oil from W86 seed, a lower amount of MDA is generated compared to the control even though it contains the highest level of easily oxidized ALA. The calculated theoretical oxidation factor based on oil fatty acid composition is the highest for W86 oil (0.910) and the lowest for control oil (0.798), with GT and W92 falling in between (0.806 and 0.871, respectively). Comparison of theoretical oxidation values with experimental results suggests the involvement of some factors protecting oils from GT, W92 and W86 plants. This hypothesis is further supported by experiments with supplementation of the control oil with commercially available α-linolenic acid in an equal and 50 % higher quantity to that found in W86 oil (Fig. 3). Indeed, control oil supplemented with α-linolenic acid oxidizes more easily than W86 oil. The results thus suggest that W86 oil contains compounds with antioxidant activity in amounts suitable for effective protection of fatty acids against oxidation.

### UPLC-PDA-MS analysis of antioxidant content

#### Water-soluble antioxidants

**Fig. 4:**
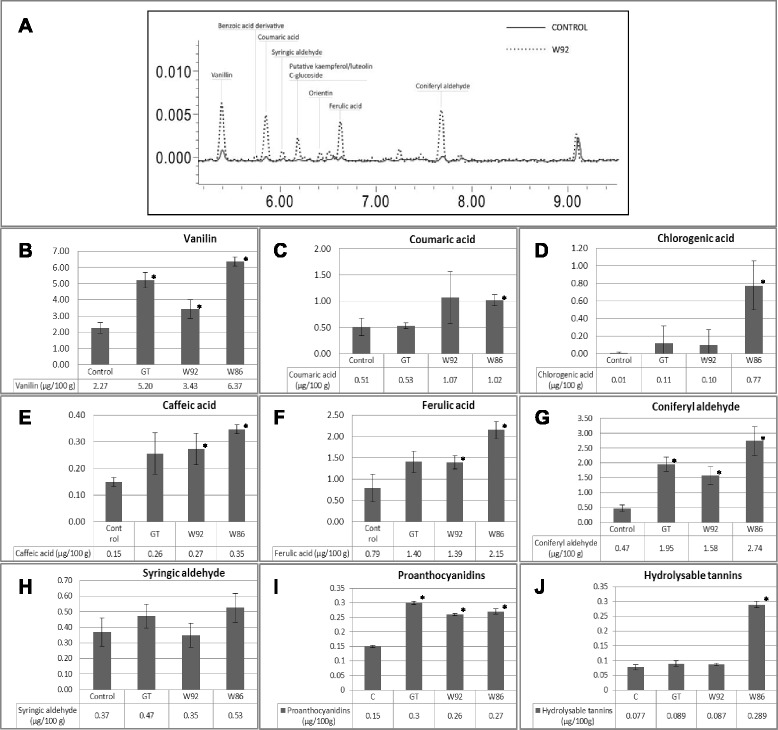
An example of UPLC analysis of methanol/water-extracted phenolic compounds from the oil of control and W92 seeds (**a**). Content (μg/100 g of oil) of the phenolic components in the oils from control and transgenic plants (**b**-**j**), measured as described in the Materials and Methods section; the results are the averages of 3 repetitions ± SD, ^*^statistically significant data (*p* < 0.05)

To identify the compounds responsible for increased oil stability in plant transgenic lines, water- and lipid-soluble antioxidant contents in the oil were determined (Table 3, Fig. 4). In all analyzed samples vanillin was identified as the most abundant phenolic compound in the flax oil. The other components are non-hydrolysable (proanthocyanidins) and hydrolysable tannins, p-coumaric acid, ferulic acid, caffeic acid, coniferyl and syringic aldehyde and small amounts of flavonoids, probably luteolin and kaempferol derivatives (Fig. 4). The ultra-performance liquid chromatography (UPLC) analysis of the oil from three transgenic lines revealed an up to threefold increase in the accumulation of total phenolic derivatives compared to the control.

**Table 3:** The content of major hydrophobic antioxidants in oil from control (Linola) and three transgenic flax lines determined by UPLC analysis

Flax oil	The content of major terpenoid antioxidants		
	Lutein (mg/100 g)	γ-Tocopherol (mg/100 g)	Plastochromanol-8 (mg/100 g)
Control	2. 99 ± 0.36	45.91 ± 4.90	40.35 ± 2.06
GT	1.59 ± 0.05*	50.08 ± 2.12	25.19 ± 3.91*
W92	1.67 ± 0.15*	43.99 ± 1.80	61.58 ± 0.51*
W86	3.24 ± 0.47	54.74 ± 2.80*	51.91 ± 1.09*

The results are the average of 3 repetitions ± SD; ^*^statistically significant data (*p* < 0.05)

The largest increase in vanillin accumulation (by about 300 %) was found in W86 oil, about 230 % in GT oil and 150 % in W92 oil. Also a large increase (by about 2-fold) in non-hydrolysable tannins for all transgenic oils was detected. The hydrolysable tannin level in the case of W86 oil was enhanced over 3-fold. The level of p-coumaric and caffeic acid was doubled in both the W92 and W86 oils. In GT oil, the p-coumaric acid level was the same as in the control oil and the increase in quantity of caffeic acid was not statistically significant. The ferulic acid level was roughly doubled (W92 and GT oil) to tripled (W86 oil), while chlorogenic acid was not detected in the control oil and was at a minimal level in GT and W92 oil (about 1 ng/g of oil). A high amount of chlorogenic acid was measured (0.77 μg/100 g oil) in W86 oil. The highest increase was observed in the case of the coniferyl aldehyde content (4-fold, 3-fold, 6-fold increase in GT, W92, W86 oils, respectively), while far smaller changes were noted for the level of syringic aldehyde (about a 2-fold increase for GT and W86 oils and a decrease for W92 oil). Also detected and measured was a benzoic acid derivative and small amounts of flavonoid C-glucosides tentatively identified as orientin, luteolin and kaempferol derivatives. Their content was higher in the oil from the transgenic plants than in the control.

To summarize, several compounds from the phenylpropanoid pathway were identified and quantified in the flax seed oil. All transgenic plants oils showed increased amounts of phenolic compounds, with the highest level detected in W86 oil.

#### Lipid-soluble antioxidants

Oil stability is also affected by lipid-soluble secondary metabolites such as tocochromanols and carotenoids, so these were assayed by liquid chromatography using a PDA detector. It was found that unrefined flax seed oils contain bioactive lipid-soluble components including lutein, plastochromanol-8 and γ-tocopherol (Table 3). In the analyzed oils, the γ-tocopherol and lutein levels were the highest in W86 oil. The highest level of plastochromanol-8 was identified in W92 oil and the lowest was found in GT oil. All of these changes were statistically significant.

### Correlation between water- and lipid-soluble antioxidant contents and oil oxidation

In order to establish the impact of the level of the two classes of antioxidants (phenolic and isoprenoid) on the oil stability, the correlation coefficients were calculated. A high positive correlation coefficient (0.75) was obtained for total phenolics content and oil stability upon exposure to 140 °C. A particularly high correlation coefficient was found for caffeic acid (1.0), ferulic acid (0.91), p-coumaric acid (0.83), chlorogenic acid (0.86) and coniferyl aldehyde (0.78). For the tannins and the most abundant phenolic, vanillin, the value of this correlation coefficient was lower (0.62 and 0.48, respectively) but still higher than for most hydrophobic components.

The correlation coefficient for oil stability and lipid-soluble components was calculated as 0.29 for γ-tocopherol and 0.49 for plastochromanol-8, while a surprisingly strong negative correlation was found for lutein content (−1.0). The correlation for sum of water and lipid soluble antioxidants and oil oxidation was 0,93 for oils at room temperature and 0,99 for 140 °C suggesting additive effect of antioxidants on oil stability.

### Antioxidant properties of secondary metabolites analyzed by DPPH method

**Fig. 5:**
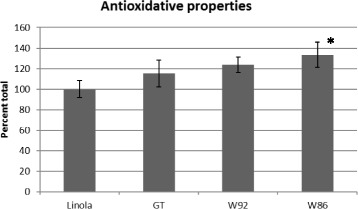
Antioxidant properties of methanol/water-extracted phenolic compounds from the oil of control (Linola) and transgenic plants (GT, W92, W86) measured by the DPPH method as described in Materials and Methods. The results (per cent control) are the average of 5 repetitions ± SD, ^*^statistically significant data (*p* < 0.05)

In order to determine the antioxidant properties of the secondary metabolites water and methanol extracted from oil, the DPPH method was used. This method is based on a single electron transfer mechanism and measures the ability of the antioxidants in oil to reduce a stable DPPH radical [26]. The level of oxidation was determined spectrophotometrically after 45 min, which allowed DPPH to react slowly with even weak antioxidants in the sample (Fig. 5). Extracts from all tested oils reduced the level of oxidation. The highest antioxidant activity was that of the extract from W86 oil (33 %), while those from GT and W92 were 23 % and 15 %, respectively.

### The impact of external compounds on oil stability

**Fig. 6:**
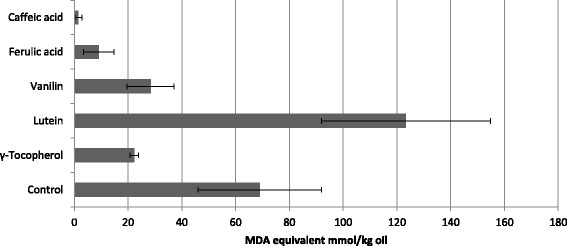
Content of fatty acid oxidation product in oil from Linola (control) flax plant and supplemented with 0.5 mM antioxidant compounds standard (marked) heated for 30 min at 140 °C, measured using the TBARS method, with data expressed as MDA equivalent (g/100 g oil). The results are the average of 5 repetitions ± SD

**Fig. 7:**
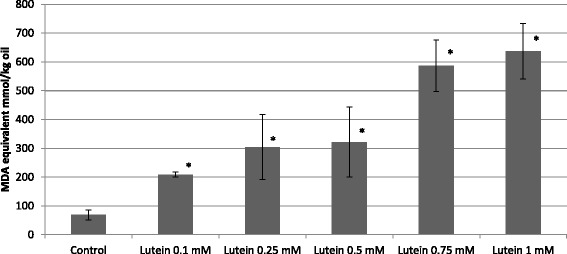
Content of fatty acid oxidation product in oil from Linola (control) flax plant and supplemented with different quantities of lutein standard. The samples heated for 30 min at 140 °C were measured using the TBARS method and data are expressed as the MDA equivalent (g/100 g oil). The results are the average of 5 repetitions ± SD, ^*^statistically significant data (*p* < 0.05)

**Fig. 8:**
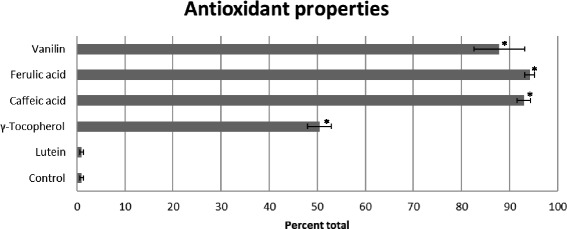
Antioxidant potential of compound standards (0.5 mM) measured by DPPH method as described in Materials and Methods. The results (per cent control) are the average of 5 repetitions ± SD, ^*^statistically significant data (*p* < 0.05)

To identify the antioxidant components most effective in oil stabilization, we supplemented the control oil with the pure compounds at a 0.5 mM concentration, and analysis was conducted using the TBARS method (Fig. 6). The components were chosen based on their high concentrations in tested oils and extreme correlations between amounts and antioxidant properties. In these experiments, we revealed that caffeic and ferulic acid were the most effective in protecting the oil from oxidation (by 94 % and 85 %, respectively). The impact of vanillin and γ-tocopherol on the level of oxidation inhibition was far lower (50 % and 70 %, respectively). It is noticeable that among the compounds identified and analyzed, only lutein acts as a pro-oxidant rather than an antioxidant. Since this effect might depend on the compound level, different concentrations (in the range from 0.1 mM to 1 mM) of this carotenoid were assayed. The result (Fig. 7) shows that lutein exhibits highly pro-oxidative activity at these concentrations, indicating that lutein itself was the source of the increased TBARS level. Next, we compared the antioxidative strength of the hydrophilic (phenolic) compounds identified in the oil with the hydrophobic compound γ-tocopherol using the DPPH method. γTocopherol is present in the water–methanol oil extract and thus might influence the DPPH measurements. The DPPH experiment revealed the great antioxidant potency of phenolic compounds such as ferulic and caffeic acids and vanillin. Far lower (about two-fold) antioxidative potential was detected for γ-tocopherol, while lutein did not show any antioxidative activity at the concentration used (Fig. 8).

## Discussion

The principal use of flaxseed in the past was industrial. Since the finding that it can provide a variety of health benefits, linseed has become established over recent decades as an important human dietary supplement and as a feed source.

Flax oil is one of the richest sources of omega-3 fatty acids and a popular addition to the diets of health conscious consumers. It is fairly well established that omega-3 fatty acids lower the risk of diseases related to cholesterol oxidation. Flaxseed is also used for poultry feeding, resulting in more nutritional eggs known as “omega-3 eggs”.

However, the high content of omega-3 fatty acids makes flax oil readily oxidized, and thus it has a minor role in the human diet. Therefore, only a limited number of cultivars with low α-linolenic acid contents (e.g. Linola) are suitable for the commercial preparation of edible oil [27], and even this oil has a very short shelf life and an inappropriate omega-3/omega-6 ratio.

In flax grains, lipids are protected against oxidation by various mechanisms, for example, the presence of antioxidants such as phenylpropanoids (flavonoids, phenolic acids), carotenoids or tocochromanols [13, 28]. The antioxidant capacity of flavonoids is related to the presence of -OH groups, which may directly bind free radicals and chelate metals [29]. By contrast, carotenoids are believed to act as free radical scavengers by electron transfer to their double-bond structure.

However, even after cold extraction, most of these mechanisms are no longer operative. Perhaps lipid-soluble antioxidants (carotenoids) are not effective enough and phenolics are not effectively extracted with oil. To avoid the rapid onset of rancidity, flax oil is often supplemented with lipid-soluble vitamins A and E (carotenoids and tocopherols) and stored in dark glass jars. As none of these protection methods are fully satisfactory, genetic engineering has been applied.

Genetic transformation of plants facilitates the controlled and rapid development of new properties and traits, in terms of both quantitation requirements and chemical composition. Nowadays, there are high expectations concerning genetic modifications of plants with changed expression of regulatory genes and those coordinating whole metabolic pathways.

A strategy to modify the synthesis of secondary metabolites involves the overexpression or repression of key genes of a given biosynthesis pathway [30]. It is often the first enzyme of the pathway that is modified. In the case of flavonoids, this is chalcone synthase (CHS). This approach (CHS suppression) was used to redirect the substrates from flavonoid biosynthesis to tannins in W86 plants [3]. However, the first enzyme may not be the rate-limiting step [31]. In such cases, it is tempting to introduce several genes of the same pathway [32]. This approach (simultaneous CHS, CHI and DFR overexpression) was used to obtain W92 flax plants, which have an increased flavonoid content [17]. A widespread modification of secondary metabolites is their glycosylation [33]. The transfer of activated monosaccharide units to an acceptor molecule catalyzed by glycosyltransferases (GTs) affects the properties of the modified molecule and its subcellular localization [34]. Glycosylation may increase a compound’s solubility in water, molecular stability and mobility [33]. This approach (GT overexpression) was used to obtain GT flax plants with increased content of glycosylated phenylpropanoids [18, 20]. Thus, three approaches have been successfully used to manipulate the content of antioxidants from the phenylpropanoid pathway in transgenic flax. Overproduction of the flavonoids aglycone, glycosylated phenylpropanoids and tannins resulted in a significant increase in their contents in flax seeds as we reported previously [3, 18, 20, 35, 36]. This strongly supports the view that the gene construct determines transgenic plant features. The phenylopropanoid increase observed in seeds is reflected in oils, however there is not a direct relationship between the phenolic contents in a flax seeds and their content in oils as a majority of flax seed phenolics are involved in a large-molecule lignan complex which is not present in flax oil. In all studied cases, the increase in phenylpropanoid contents resulted in the expected enhancement of the antioxidant properties of the oil extract and thus an increase in oil stability.

Elevated antioxidant level in transgenic oils improved their shelf life assessed by concentration of primary and secondary products in comparison to control. During the 6 months of storage which is a recommended period for high PUFA oils negligible changes in oxidation status of transgenic oils were noticed while in Linola a significant increase in formation of hydroperoxides was observed. Shelf life prediction from DSC analyses revealed that W86 antioxidant composition was the most effective in protection against oxidation induction of oil [24, 37]. However W92 had the longest propagation period of oxidation among the studied oils. This indicated that the elevated flavonoid contents vigorously trapped once formed radicals extending the propagation time [37, 38]. Compared to the control, the highest increase in stability, measured by MDA content, was detected for the oil from plants overproducing flavonoids (W92). The medium increase was for oil from plants over-accumulating glycosylated derivatives (GT). The lowest, but still significant, was for those overproducing tannins (W86). These data fairly well concurred with those derived from other studies that described analyses of oil oxidation in relation to their PUFA content. As seen from studies of the peroxidation of lipid standards, an increasing number of unsaturated C = C bonds increases the susceptibility to oxidation [27]. Thus, it was observed that higher PUFA levels resulted in easier oil oxidation. However, it is interesting to note that of the investigated oils, that from the W86 plant seeds, although richest in omega-3 fatty acids, is the most stable when exposed to high temperature. This suggests that the lowered stability caused by a high PUFA content may be overcome when a high enough level of effective antioxidants is present. It is obvious that the antioxidant content in the oil mainly determines its susceptibility to oxidation at high temperature. The lower antioxidant activity of glycosylated phenolic compounds when compared to the compounds possessing free hydroxyl groups has been previously shown and may be related to the lower stability of GT oil [39].

To study the factors influencing the stability of flax oil, the antioxidative components of the oils from the control and transgenic flax were determined. From the phenylpropanoid pathway, vanillin, tannins, p-coumaric, chlorogenic, caffeic, ferulic acid, coniferyl and syringic aldehyde were identified. The terpenoid pathway was represented by plastochromanol-8, tocopherol and lutein compounds. It should be pointed out that this is the first report with a detailed analysis of the phenolic compounds in flax oil. Previously, phenolic compounds in plant oils were assayed mainly with the Folin-Ciocalteu method, which does not identify constituents and only roughly informs about the phenolic content [40, 41]. When assayed by high-performance liquid chromatography, few compounds were identified, and in the case of flax, vanillin was the only phenolic detected [42]. Published data on phenolic content and the antioxidative properties of other plant oils analyzed by the DPPH or TBARS method are also quite imprecise, and quite often contradictory [40–43]. Of the studied transgenic plants, the highest quantity of total phenolics was found in W86 oil, the second highest in GT oil, and the lowest in W92 oil. It is remarkable that the control oil contains a far lower quantity of phenolics than that from the transgenic plants. The data from the phenolic measurements fairly well correlate with oil stability. This is in contrast to the terpenoid compound content, where no such correlation was found, but there is no doubt that these compounds affect the total antioxidant capacity of the oil as well. In addition to preventing rancidity, both types of antioxidants (phenylpropanoids and terpenoids) could increase the commercial value of food products based on flax oil and might be beneficial for human health. When consumed together with PUFA, they can reduce the risk of various diseases [44].

It is desirable to identify the compounds that most efficiently protect lipids against oxidation and subsequently modify their content via genetic engineering. The analysis of the antioxidant properties of various compounds from both the phenylpropanoid and terpenoid pathways using the DPPH and TBARS methods revealed that phenolic acids have the highest antioxidant capacity in radical scavenging as well as in the inhibition of the formation of secondary oxidation products, respectively. The general conclusion that can be drawn from this experiment is that water-soluble compounds, such as phenolic acids, are more effective in protecting fatty acids against oxidation than lipid-soluble compounds, such as tocopherol and lutein. It is interesting to note that lutein shows pro-oxidative activity at high temperature, which was further confirmed in the in vitro experiment. The pro-oxidant activity of lutein in oil has been previously shown both in the dark and in the light, especially at higher temperature, and this may be explained by lutein instability against heat leading to the formation of degradation compounds acting as oxidation promoters [45]. The result obtained clearly showed a pro-oxidative, concentration-dependent effect of lutein on fatty acid oxidation. In our previous report, the potential to reduce peroxidation was tested using natural antioxidants (carotene and quercetin) at concentrations ranging from 10 to 250 mM [8]. The formation of TBARS was most efficiently reduced by 25 mM concentrations of both carotene and quercetin. Higher concentrations of carotene increased the level of TBARS. Thus, the efficiency of antioxidants varied depending on their chemical nature and concentration [46]. In general, the scavenging ability of phenolic acids is dependent on their electron-donating ability and inhibition of lipid peroxidation induced by superoxide. Tocopherols usually trap the hydroperoxide intermediates and stop the autoxidation chain reaction whereas carotenoids act as scavenger of singlet oxygen [41, 42]. Tocopherols in contrast to phenolic acids has been shown to be more effective at a higher temperature than at a lower temperature [47, 48] but in the same time γ-tocopherol degrades in high temperatures more rapidly than phenolics or plastochromanol–8 [49]. In the other hand higher temperature did not negative influence on effectiveness of ferrulic acid on the inhibition of initiation radical formation. Ferrulic acid is one of powerful antioxidant investigated regardless of temperature and probably may stabilize γ-tocopherol from high temperature destruction. The higher thermal stability of phenolic compounds then tocopherols and carotenoids seems to be important factor influencing better stability of phenol-rich oils in accelerated oxidation tests (TBARS and DSC) [50]. However, the synergistic action cannot be discounted as combinations of phenolic compounds and tocopherols may increase its antioxidant properties because they stabilize each other. Phenolics compounds were reported to act as stabilizators of tocopherols [51] and next tocopherols can protect and regenerate carotenoids [52].

## Conclusion

The general conclusion from this study is that water-soluble rather than lipid-soluble antioxidants are more suitable for fatty acid protection against oxidation, with phenolic acids being the most effective of the water-soluble antioxidants but the lipid-soluble antioxidants participate in general protection. Furthermore, the engineering of the phenylpropanoid pathway in flax is beneficial for flax oil stability, and the extent of lipid protection depends on the total antioxidants concentration.

## Methods

### Plant materials

The transgenic flax plants were previously obtained by agrotransformation of the Linola cultivar. W92 was generated using a triple gene construct containing *Petunia hybrida* cDNA coding for chalcone synthase (CHS, EMBL/GenBank database acc. no. X04080), chalcone isomerase (CHI, EMBL/GenBank database acc. no. X14589) and dihydroflavonol 4-reductase (DFR, EMBL/GenBank database acc. no. X15537) in the sense orientation and under the control of the strong and non-specific 35S CaMV promoter and OCS terminator [17]. In W86, down-regulation of the endogenous chalcone synthase gene was achieved by overexpression of cDNA of the petunia homologue (CHS, EMBL/GenBank database acc. no. X04080), probably via the siRNA mechanism [3]. For GT, the construct containing the 7-O-glycosyltransferase gene (SsGT1, EMBL/GenBank database accession no. AY033489) derived from *Solanum sogarandinum* was used in the sense orientation under the control of the seed-specific NAP (napin) promoter and OCS terminator [18].

Linola flax seeds (control) were obtained from the Flax and Hemp Collection of the Institute of Natural Fibres, Poland. We used the fourth generation of field-grown transgenic plants (W92, W86, GT types) from the 2011 growing season throughout this study. Plants were harvested after 4 months’ growth.

### Preparation of oil from seeds

25 kg of flax seeds from field-grown plants were ground and transferred to an industrial worm gear oil press (Oil PressDD85G – IBG Monoforts Oekotec GmbH& Co). This is a typical industrial method for cold pressing of oil. The obtained oil was stored at 4 °C under nitrogen (N2) until needed. All the precautionary measures were undertook to limit exposition of oil being pressed to light. The oils were then stored in amber glass bottles until needed.

### Conversion of fatty acids into fatty acid methyl esters

Prior to GC analysis, the fatty acids were converted into their methyl esters (FAME). 5 μl of oil was placed in a glass tube with a Teflon-sealed screw cap and supplemented with 500 μg of pentadecanoic acid as an internal standard as described before [53].

Each sample was made in triplicate. 1 ml of 0.5 M KOH in anhydrous methanol was added to each sample, which was shaken well and incubated for 30 min at 70 °C. After cooling, 1 ml of 1.25 M HCl in anhydrous methanol was added and the sample was again mixed and incubated at 70 °C for 30 min. Samples were cooled, and 1 ml of hexane and 3 ml of saturated NaCl were added to each. Samples were then mixed well for 5 min and the hexane fraction containing FAME was collected into fresh Eppendorf tubes. Extraction was repeated by adding an additional 1 ml of hexane, and the collected hexane fractions were stored at 4 °C until measurement (at the latest on the next day).

### FAME analysis

The fatty acid content was measured on an Agilent 7890A gas chromatograph with an FID detector on an Omegawax 250 column (30 m x 0.25 mm x 0.25 μm) using the program developed in our laboratory: injector temperature: 250 °C; split 100:1; gas pressure 17.81 psi; carrier gas: nitrogen; Owen program: 140 °C (hold 2 min) to 240 °C (4 °C/min; hold 10 min); FID detector temperature: 260 °C. Before the analysis, a series of fatty acid standards (Sigma-Aldrich) was run to determine the retention times for each compound. Each sample was esterified and measured in 3 replicates.

### Shelf life studies

Official methods were used for the determination AV, PV, pAV, CD and CT contents (CEN, 2008, 2009, 2010, 2002) during shelf life of oils.

The shelf life assessment was also performed by a Perkin Elmer differential scanning calorimeter Pyris 6. The equipment was calibrated using pure indum in a hermetically sealed aluminium pan. The oil sample (5–6 mg) was placed in open aluminium pan filled with sodium chloride (ca. 0.01 g). The open aluminium pan filled with equal amount of sodium chloride was used as the reference. The isothermal temperature was programmed at 100, 110, and 120 °C and air was passed through the sample at 20 ml/min. The DSC parameters (t0 – the onset time of the oxidation reaction, tmax – time at the end of propagation period, regression equation) were calculated using Pyris 6 software package. A kinetic rate constant was taken as the inverse of the onset time [24].

### TBARS measurements

The TBARS (thiobarbituric acid reactive substance) assay is a widely used method for measuring lipid peroxidation. [8]. The key of this assay is to measure the level of substances that react with thiobarbituric acid. MDA dominates the pool of substances that react with TBA. Thus results presented in this paper are reported as the equivalent of MDA, a compound that results from decomposition of polyunsaturated fatty acid lipid peroxides. Oil samples (4 μl) were oxidized at 140 °C for 30 min in tightly closed glass test tubes in a laboratory oven. After the initial baking time, 2 ml of reagent (15 % TCA and 0.37 % TBA in 0.25 M HCl) was added to each sample, and the mixture was thoroughly mixed. Then, the test tubes were heated at 100 °C for 15 min and cooled under running tap water. After a 10-min centrifugation, the absorbance at 535 nm was measured. To determine the influence of the added extracts and standards on oil protection, 4 μl of extracts was added to 4 μl of oil samples and mixed before oxidation at 140 °C. For each oil, 5 repetitions of the measurement were performed in one experiment. The whole analysis was repeated independently at 3 different times. For final calculation of TBARS, a standard curve of MDA was made to present the results as equivalents of MDA (g/100 g oil).

### Extraction of hydrophilic components from oil

Water-soluble compounds were extracted from 10 ml samples of oil using a method developed in our laboratory. The oil was solubilized in 20 ml of n-hexane and then extracted three times with 10 ml of 80 % methanol/water in an ultrasonic bath for 15 min. The extract was centrifuged at 5500 rpm for 10 min. The aqueous methanol phase was collected and combined, and the extracts were concentrated in an N2 stream and resuspended in methanol. The samples were then filtered through a 0.22-μm Acrodisc filter and stored at 20 °C until needed.

### UPLC-PDA-MS analysis of hydrophilic components from oil

Methanol- and water-extracted phenolic compounds from the oil were measured using UPLC combined with two detectors (PDA and MS). Simultaneous use of two techniques (UPLC-PDA-MS) allows us to obtain more detailed data. The flax seed oil extracts were analyzed on a Waters Acquity UPLC System with a 2996 PDA detector and Waters Xevo QTof MS System mass spectrometer, using Acquity UPLC column BEH C18, 2.1 x 100 mm x 1.7 μm as described previously [35] with slight modifications. The mobile phase was composed of solvent A (0.1 % ammonium formate, pH 3) and solvent B (100 % acetonitrile) in a gradient flow: 95 % A/5 % B at 1 min, 2–12 min gradient to 70 % A/30 % B; 13–20 min gradient to 0 % A/100 % B; and 21 min gradient at 95 % A/5 % B with a 0.4 ml/min flow rate. The MS spectra were recorded in ESI positive mode for 13 min in the 50–800 Da range. The parameters were: nitrogen flow 800 l/h, source temperature 70 °C, desolvation temperature cone 400 °C, capillary voltage 3.50 V, sampling cone 30 V, cone voltage ramp 40–60 V, scan time 0.2 s. The identity of the components was determined on the basis of retention time and UV and mass spectra of authentic standards. The program described was followed by washing and reconditioning of the column. The flow rate was 0.4 ml/min. The compounds’ spectra were measured at 280 nm. The identification and quantification were done by comparison with authentic standards.

### Measurement of proanthocyanins

For the measurements, methanol- and water-extracted phenolic compounds from the oil (10 ml) were used. The extract was centrifuged, evaporated to near dryness at 40 °C under a vacuum and then proanthocyanins were hydrolyzed with 1 ml of n-butanol/HCl (95:5, *v/v*) and 33 μl of 2 % (*w/v*) NH4Fe(SO4)2 12H2O in 2 M HCl for 40 min at 95 °C. The extract was centrifuged at 14 000 g for 10 min, and the supernatant was used for proanthocyanin content evaluation. Proanthocyanin detection was carried out by measuring absorption at 540 nm, and compound content was expressed as catechin equivalents [54].

### Measurement of hydrolysable tannins

For the measurements, methanol- and water-extracted phenolic compounds from the oil (10 ml) were used. The extract was applied onto a column (1 × 10 cm) packed with Sephadex LH-20 gel and eluted with ethanol (25 ml). This fraction mainly contained low molecular weight phenolic compounds including monomers, and a part of dimers and trimers of hydrolysable tannins and procyanidins was discarded. Subsequently, acetone:water (1:1, *v/v*) solution (30 mL) was used to elute the hydrolysable tannins. The acetone was removed using a rotary evaporator, and the aqueous residue was condensed in a vacuum. The phenolic group content was measured using the Prussian blue method [55], and the hydrolysable tannin content was expressed as gallic acid equivalents.

### UPLC-PDA analysis of lipid-soluble components from oil

The lipid-soluble compound content of the flax oils was determined using a Waters Acquity UPLC System with a 2996 PDA detector using a method developed in our laboratory. A 20 μl sample of flax seed oil was dissolved with 200 μl of acetone, vortexed and left at 4 °C. Next, the samples were adjusted to 1 ml with a mixture of acetonitrile/methanol (1:1) and shaken for 1 min. This solution was directly injected onto a UPLC BEH C18, 2.1 × 100 mm, 1.7 μm column after filtration through an Acrodisc 0.22 μm filter (Gelman Sciences, Ann Arbor, MI). The mobile phase for tocopherol and lutein detection was composed of solvent A (methanol) and solvent B (acetonitrile) in a gradient flow: 1–6 min to 10 % A/90 % B; 7 min to 0 % A/100 % B, 8 min to 100 % A/0 % B, and 9–10 min to 10 % A/90 % B. The mobile phase for plastochromanol-8 detection was composed of solvent A (methanol) in an isocratic flow: 8 min at 100 % A. The program was followed by washing and reconditioning of the column. The flow rate was 0.4 ml/min for tocopherol and lutein and 0.7 ml/min for plastochromanol-8 detection. The runs were monitored at 200–500 nm and the peaks were integrated for tocopherols and plastochromanol-8 at 290 nm and for lutein at 445 nm. The identification and quantification was done by comparison with authentic standards.

### DPPH measurement of oil extract

The DPPH (1,1-diphenyl-2-picrylhydrazyl) free radical-scavenging capacity of the methanol/water extract from the oil was evaluated with the DPPH stable radical. Method [41].

Briefly, a fresh 0.1 mM solution of DPPH in methanol was prepared and 1 ml of this solution was added to 50 μl of oil extract and allowed to react in the dark at room temperature. The concentration of DPPH was determined spectrophotometrically after 45 min at 517 nm against the blank. The DPPH value was calculated using the formula: 1-(A/C)100 %, where A means the sample and C is the control without added extract or standard.

### Theoretical oxidation factor of oil

Based on the fatty acid composition of each oil sample the mathematical value of the FA oxidation factor was calculated according to the equation:

$$ \mathrm{FAox}\kern.5em \mathrm{factor}=\left(0.02,\times, \mathrm{C}18:1+1\times \mathrm{C}18:2\times \mathrm{C}18:3\right)/100 $$

The value of the coefficient is the mathematically calculated factor of oxidability of FA based on experimental studies on susceptibility to oxidation of fatty acids [56]. In accordance with the oil rich in unsaturated fatty acids having a lot of easily oxidized double bonds, the value of the coefficient will have increased.

### Statistical analysis

The data obtained were statistically analyzed using the t-test for independent samples and STATISTICA 10 software (StatSoft, Poland). The results were statistically significant at *p* < 0.05.
